# Non-Instrumental Test for the Evaluation of Tongue Function

**DOI:** 10.7759/cureus.18333

**Published:** 2021-09-27

**Authors:** Bruno Bordoni, Allan R Escher

**Affiliations:** 1 Physical Medicine and Rehabilitation, Foundation Don Carlo Gnocchi, Milan, ITA; 2 Anesthesiology/Pain Medicine, H. Lee Moffitt Cancer Center and Research Institute, Tampa, USA

**Keywords:** oral cancer, manual therapy, physiotherapy, myofascial, fascia, osteopathic, tongue

## Abstract

The tongue undergoes various negative adaptations in the presence of local or systemic pathologies, adversely its behavior within the body context. Tongue assessments to correctly diagnose its functions are carried out using instrumentation, such as ultrasonography, magnetic resonance imaging, electromyography and different intraoral devices (swallowing, strength, posture, phonesis). Currently, there is no dynamic non-instrumental test in the scientific literature to highlight any lingual dysfunctions. The article describes a non-instrumental test for the assessment of lingual function in the body context, to obtain preliminary information on the quality of the neurological activities of the tongue, with respect to the balance and muscle strength that the patient expresses. The text briefly reviews the anatomy of the tongue and describes a clinical case to better understand how to use this test. Further studies will be needed for the validation of the test.

## Introduction

The lingual muscle complex represents a contractile organ that has multiple functions and anatomo-neurological characteristics that are unique in the human body. The embryological origin of the tongue derives from the pharyngeal arches, and in particular, the connective tissue and the blood and lymphatic vessels arise from the ectoderm (neural crests), while the muscle tissue is formed from the mesoderm (occipital somites) [1]. The first action of the tongue during its formation is swallowing, in about the third month of pregnancy [2].

The muscles that make up the tongue and allow the expression of different functions with different functional morphologies are divided into extrinsic (eight pairs of muscles) and intrinsic (eight pairs of muscles), for a total of 16 contractile districts [2]. The tongue is able to act as a muscle for breathing, speech, swallowing, body movement, and body posture [3]. Based on the function for which it must intervene, the movement it performs is specific to the action for which it is called to act. To give an example, during a eupnoeic act, the tongue undergoes an anterior thrust of its inferior and posterior area and, at the same time, its posterior-superior tract makes a caudal and posterior movement [4].

The innervation of the tongue involves different nerve pathways. The IX or glossopharyngeal cranial nerve is responsible for the lingual gustatory capacity, in particular for the posterior third of the tongue; the nerve itself supplies parasympathetic fibers [5]. The glossopharyngeal nerve anastomoses within the lingual complex, with the lingual nerve (mandibular branch of the trigeminal nerve), with the vagus nerve or X cranial nerve, and with the hypoglossal or XII cranial nerve [6]. The XII nerve guides the muscle activity of most of the contractile districts, while the X nerve, in addition to giving parasympathetic fibers, is activated by the contraction of the palatoglossus muscle (an extrinsic muscle of the tongue) [7,8]. The lingual nerve innervates the anterior portion for gustatory and somatic sensitivity and anastomoses with afferent fibers of the cranial facial nerve or VII (cord of the eardrum) [9]. Sympathetic fibers arrive at the lingual musculature from the superior cervical ganglion [10].

The tongue influences balance and body movements, as well as the muscular strength of the skeletal muscles, through a close neurological relationship between the tongue and the central nervous system [3]. To give an example, tongue stimulation sends multiple signals from the trigeminal and vagal pathways, involving the cerebellum and vestibular nuclei (balance-processing network) [11]. If such areas are injured, regardless of the cause, the patient's ability to manage body movement (balance and muscle strength) is decreased, while, if the tongue of such patients is stimulated (external electrical equipment), balance and strength expressed improve [11]. If these neurological relationships are functional, the tongue does not disturb the body's somatic system, or, at least, the lingual afferents are effective for maintaining central and peripheral neurological expressions. The article describes a non-instrumental test for the assessment of lingual function in the body context, to obtain preliminary information on the quality of the neurological activities of the tongue, with respect to the balance and muscle strength that the patient expresses. The test is called the Performance Tongue Test (PTT).

## Technical report

**Figure 1:**
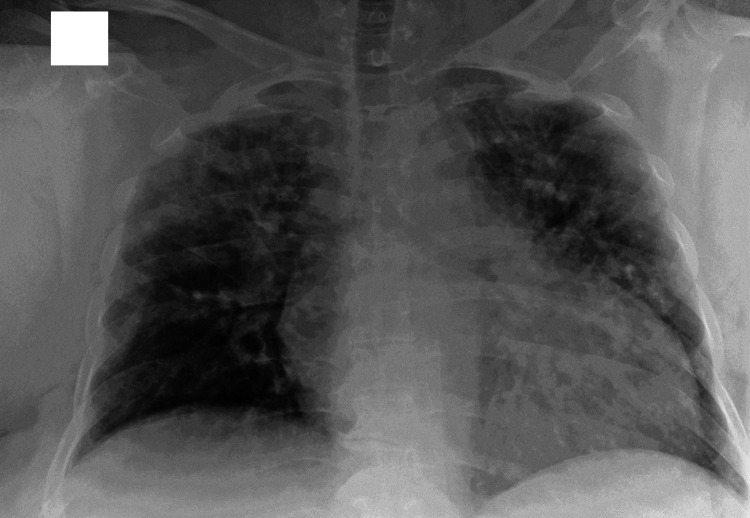
The radiographic examination demonstrates a widespread increase in the peribroncovascular interstitial texture with associated multiple parenchymal thickening areas arranged in correspondence with the upper field of both lungs. Heart increased in volume; sinus costophrenics hypo-expanded but free from effusion. The x-ray picture confirms the presence of bilateral interstitial pneumonia strongly suspected for a positivity to COVID-19.

To better understand how to use the PTT, we will present a clinical case in a context of osteopathic manual medicine, where the test made it possible to direct the clinician towards treatment for the most in need of the anatomical area. A 60-year-old obese patient (body mass index 29,411kg/m2 - 1,70 body height, 85 kilograms), with no known cardiological and pulmonological history, type 2 diabetes in oral therapy, with worsening dyspnea and in the presence of pyresis was admitted to our pulmonology ward. The electrocardiogram revealed an atrial fibrillation with a rapid ventricular response (RVR) and, through thoracic auscultation, a bilaterally absent vesicular murmur was reported. The X-ray taken showed dilated cardiomyopathy (25% ejection fraction after echocardium), and the presence of bilateral interstitial pneumonia; the molecular swab was positive for Covid-19 (Figure 1).

**Figure 2:**
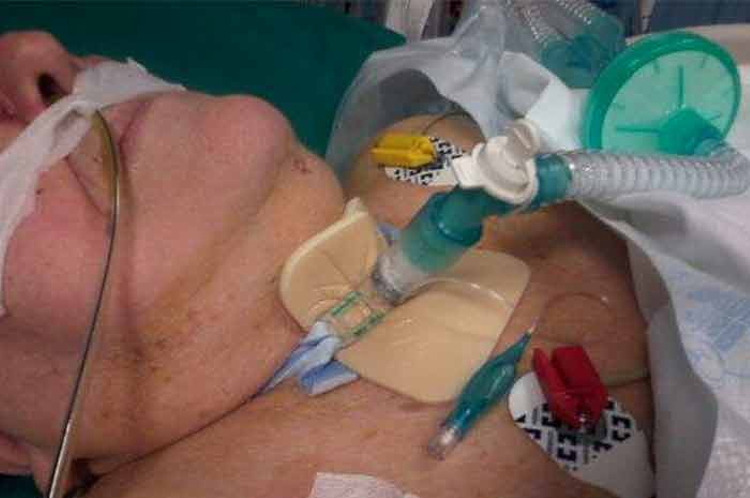
The patient shows the result of a percutaneous tracheostomy, an opening at the level of the 2nd-3rd cartilaginous ring of the trachea, inside which a cannula is inserted that creates direct communication between ambient air and lower airways, reduces the anatomical dead space and improves lung ventilation.

Since the respiratory clinical picture was worsening (SaO2 of 85% on a non-rebreather mask), the surgeon decided to have the patient undergo a surgical procedure of tracheostomy (presence of phlegm obstructing the right main bronchus), and to keep the patient in sedation (for one week) (Figure 2). One week after awakening and with stabilized clinical values, the surgeon removed the cannula.

The same patient was transferred after a month of monitoring and intensive care to the cardiopneumological rehabilitation department of the same hospital; his drug therapy upon arrival in rehabilitation was composed as follows: omeprazole 20 milligrams, bisoprolol 5 milligrams for two, Eliquis 5 milligrams for two, Entresto 24/26 milligrams 1 tablet for two, Aldactone 25 milligrams, allopurinol 300 milligrams, Lanoxin 0.25 milligrams, Lasix 25 milligrams, metformin 500 milligrams three times. Walking saturation was 97%, atrial fibrillation was resolved by pharmacological cardioversion. The follow-up examination with computed tomography shows few areas of ground glass subpleural and basal hyperdensity, mainly in the upper and posterior fields. Repetition of the echocardiographic examination shows an improvement in the frequency of ejection (35%).

The patient's rehabilitation goal is to regain full motor and respiratory autonomy after one month of hospitalization and limitations in autonomous movement. The patient's usual rehabilitation process consisted of bodyweight exercises, exercises with respiratory instruments (stimulators of inhalation against resistance), and cyclette exercise for up to 30 minutes twice a day. The extent of the workload was always gradual and always respecting the performance capacity of the patient and the clinical picture. The patient, despite the negative electromyographic tests, shows persistent muscle weakness in the breath tests (via portable spirometry and the inspiration stimulator or coach), the ability to get up from the chair, and the maintenance of posture in an upright position.

**Figure 3:**
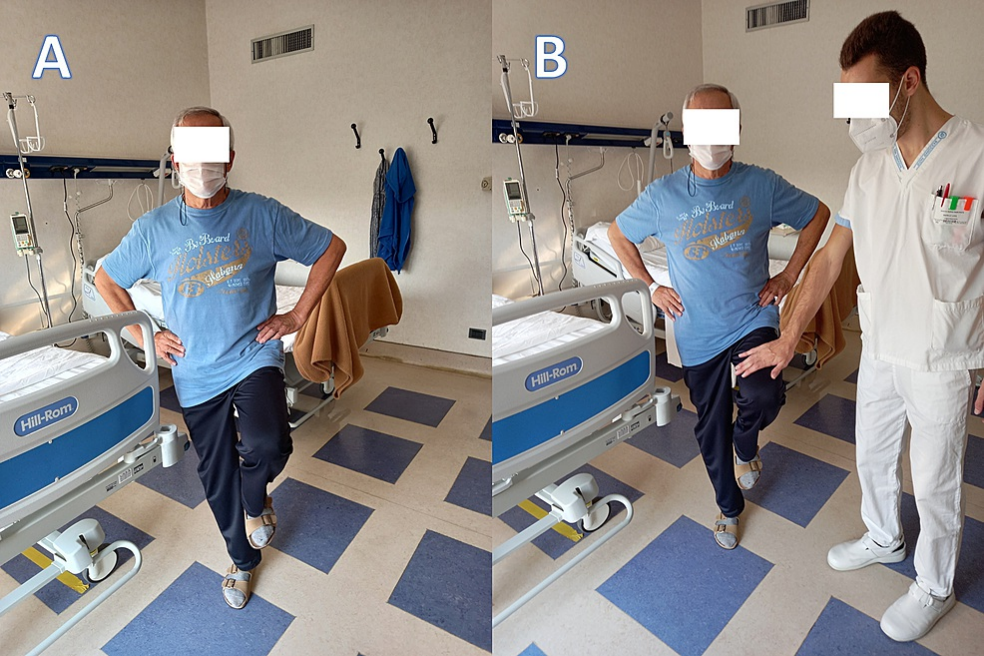
Figure A illustrates the patient raising the thigh during the first phase of the PTT, and with the tongue against the palatine spot (not visible in the photo); figure B illustrates the second phase of the test, that is, the lifting of the thigh against the resistance of an operator and the tongue against the palatine spot (in the photo it is not visible).

The visit by the osteopathic clinician highlighted a functional limitation of the diaphragm muscle (through palpation of the diaphragmatic area), and a weakness of lingual strength (through manual evaluation). The patient showed no dysphagia, dysfunction in phonesis or sleep apnea (via instrumental evaluations such as polysomnography). Manual evaluation of the osteopathic clinician was derived from existing literature [4,6]. To discriminate which anatomical area between the diaphragm and the tongue was the most dysfunctional, and capable of negatively affecting the body system, the osteopath subjected the patient to two tests. The first to verify the function of the diaphragm muscle, through the Bordoni Diaphragmatic Test, which was negative [12]. The second test to verify the lingual function, that is, the PTT, gave a positive result. To perform the PTT, the patient is asked to stand for a few seconds. The patient is asked to place his hands on his hips while performing two alternating flexions of the thighs (one knee flexed upwards and vice versa, for a total of four movements). After waiting a few seconds, the patient is asked to repeat the actions, but this time placing the tongue against the palatine spot, posterior to the two upper incisors. Generally, the tongue at rest is placed behind the palatine spot without any lingual push, but in the test the patient is asked to consciously push the tongue against this oral area. If the patient's balance improves, the information that the tongue sends and that the brain processes are correct; if the balance felt by the patient does not change, the test is positive, that is, the tongue does not cooperate with the central nervous system. To have a counter-proof of the result obtained, the patient is asked to perform the same previous actions, but without consciously placing the tongue against the palatine spot; an operator next to or in front of the patient will put his hand on the patient's thighs during the individual movements, placing a slight counter-resistance or force to be overcome. After a few seconds of rest, the same actions are carried out by the patient and the operator, but the patient is asked to push the tongue against the palatine spot. The test is negative if the strength demonstrated by the patient during movement and with the tongue held voluntarily against the palatine spot is increased, otherwise, if the strength does not change or decreases, the test is positive. The PTT is built in two phases and with four sections (Figure 3).

In the clinical case in question, the test proved positive, as both the balance and muscle strength shown decreased when the patient was asked to consciously place his tongue against the palatine spot. The osteopath worked the tongue manually, leaving out the diaphragmatic area. The treatment consisted of a gentle approach, referred to as the unwinding technique (osteopathic technique), where the operator waits for the tongue to start expressing homogeneous involuntary movements. After two treatment sessions, in two days, the patient improved his ability to get up from the chair without assistance, and improved his ability to maintain an upright position, with the benefit of autonomy. The portable oximeter and the coach showed an increase in the strength of the breath (in oxygenation with a 95% SaO2). By repeating the PTT, the test always turns out to be negative. After a week of further functional rehabilitation, the patient was discharged in perfect autonomy. Drug therapy for patient discharge was as follows: omeprazole 20 milligrams, bisoprolol 5 milligrams for two, Eliquis 5 milligrams for two, Entresto 24/26milligrams 1 tablet for two, Aldactone 25 milligrams, allopurinol 100 milligrams, metformin 500 milligrams three times.

## Discussion

There are many pathological contexts that negatively affect the lingual function. To give some examples, after a surgical treatment of resection of a lingual tumor and possible reconstruction of the contractile tissue, a dysfunction of the tongue remains; there is a lower expressed force (maximum tongue pressure: MTP) and alteration of swallowing (dysphagia), there is an altered resting position of the tongue with an increased finding of sleep apnea. In the presence of Parkinson's disease, the lingual complex demonstrates a decrease in contractile force, which leads to dysphagia, disturbances in the correct emission of phonemes and the presence of sleep apnea syndrome. The presence of a taste disorder (hypogeusia or ageusia) alters the mechanical properties of the lingual receptors. We know that the vibrations generated by the mandible or the submandibular muscles activate the extrinsic muscles of the tongue through the mechanoreceptors, and this mechanism improves the reflexes related to the opening of the upper airways; we can assume that a dysfunction of these receptors can induce sleep apnea. Patients suffering from neuromuscular disorders have different lingual dysfunction, such as a tongue enlarged in patients with Duchenne muscular dystrophy and myotonic dystrophy type 1, or a complex lingual atrophic in patients with amyotrophic lateral sclerosis. The percentage of dysphagia, syndromes of sleep apnea, alteration of the coordination of the muscles of the tongue is very common in this pathological sphere; the loss of lingual function correlates with the severity of the disease. The alteration of the function of the lingual system leads to systemic consequences, such as malnutrition, cardiovascular and cerebral pathologies, behavioral alterations linked to the decreased ability to communicate. Knowing if the tongue negatively affects the body context or is influenced by other anatomical areas subject to pathology, becomes essential to choose the most suitable curative approach. There are different tools for evaluating the tongue, such as ultrasonography, magnetic resonance, electromyography and different intraoral devices (swallowing, strength, posture, phonesis) [13,14]. The PTT represents the first non-instrumental test for the functional evaluation of the tongue, regardless of the pathological context. The test arises from anatomical and neurological considerations and from research carried out on patients with balance disorders (or simply elderly subjects). We know that by placing the tongue against the palatine spot during active resistance movements, in healthy subjects, the recorded force increases [3]. Probably, this action stimulates the exteroceptors residing in the area of the spot. These exteroceptors are connected to the trigeminal nerve (nasopalatine nerve), which sends the information collected to the brain stem for the trigeminal nuclei, the reticular substance, the cortical and cerebellar area; these areas are related to movement and posture [14]. If these relationships are altered, for example, the tongue does not move correctly, the palatal exteroceptors do not send correct afferents, and postural problems may arise [14]. In the healthy elderly person, it seems that these palatal receptors undergo an increase in the activation threshold, decreasing the effect of the tongue on the strength-posture of the body [15]. The tip of the tongue itself is rich in mechanoreceptors, with a sensitivity equal to that of the fingers; the stimulation of these receptors allows a correct posture of the occipito-cervical tract for adequate swallowing or phonation [16]. The tongue, with a rich presence of multiple mechanosensitive receptors with different properties, is an effective interface with the central nervous system [17]. Movement stimulates these receptors, activating the cerebral and cerebellar areas to control posture, strength, and gait stability [17,18]. In addition, the stimulation of the motor activity of the tongue involves the neurological pathways that manage visual information, adding sensory integration for maintaining balance and movement [19,20].

PTT has limitations. The test cannot be performed by uncooperative patients, as for the presence of motor and/or cognitive degenerative pathologies, as well as for other pathologies or acute traumas that do not allow the voluntary movement of the limbs and the tongue. We must remember that the PTT is not a diagnostic tool, but a tool for obtaining preliminary information, which will require further specific evaluations, as for all non-instrumental tests. Further studies will have to be carried out for the validation of the PTT.

## Conclusions

The tongue is able to influence the vestibular, cortical and cerebellar areas that manage information for the expression of the movement of skeletal muscles, which are essential for balance and strength. The article describes for the first time a non-instrumental test for the assessment of lingual function, referred to as the Performance Tongue Test or PTT. The test is not diagnostic, but provides useful information to help the clinician to further specific investigations and, possibly, to discriminate the patient's symptoms more quickly. Further studies will be required for the validation of this new test.
